# Uncovering eutrophication dynamics using PCA-driven spatiotemporal assessment of lake water quality indicators

**DOI:** 10.1007/s10661-026-15712-4

**Published:** 2026-08-21

**Authors:** Sujan Timilshina, Suresh Sharma, Abishkar Paneru, Matthew Davis, Thomas Mathis

**Affiliations:** 1 Civil and Environmental Engineering, Youngstown State University, 1 Tressel Way, Youngstown, OH 44555 USA; 2 Youngstown State University, 1 Tressel Way, Youngstown, OH 44555 USA; 3 DSI, LLC, Edmonds, WA USA

**Keywords:** Principal Component Analysis (PCA), Correlation, Water quality parameters, Blue-green algae (BGA), Stratification, Eutrophication

## Abstract

The degradation of freshwater ecosystems due to nutrient enrichment has underscored the imperative for robust water quality assessment. In this study, 11 monitoring stations were established in the critical water supply reservoir, Tappan Lake, Ohio, USA, to record the key physicochemical and biological parameters on a biweekly basis, especially in the summer. The parameters like temperature, pH, dissolved oxygen (DO), turbidity, conductivity, and biological parameters like chlorophyll-a, blue-green algae (BGA), were analyzed to identify spatiotemporal variations and latent patterns in water quality. More than twenty-three principal component analyses (PCA) were employed on high-resolution, multi-temporal datasets of the lake to investigate lake water quality dynamics. By and large, PCA results suggested that the first two principal components collectively represent nearly 70% of total variance, with a factor loading for temperature (0.818), Chl-a (0.740), and BGA (0.949) emerging as the most influential parameters, jointly accounting for a substantial proportion of variance across principal components. Temporally, the first principal component represented 63% in spring, 55% in summer, and 62% in fall, indicating the dynamic nature of the lake's ecosystem and biological activity. These indicators revealed a strong association of eutrophication dynamics with temporal period, underscoring them as primary ecological stressing patterns. Vertical stratification phenomena and distinct temporal clustering were detected, indicating the interplay between thermal structure and biogeochemical cycling. By isolating the dominant patterns of water quality depletion, this research offers insights into water quality nuances for informed decisions and the targeted management interventions for the lake restoration and management of mid-size reservoirs.

## Introduction

Freshwater ecosystems have been facing escalating threats driven by both climatic variability and intense anthropogenic activities across the world (Carpenter et al., 1998). Consequently, lakes and reservoirs, which serve as a critical source for potable water supply, recreation, and ecological activities, are undergoing alarming deterioration in water quality (Bhateria & Jain 2016; Ho, 2019). The decline is primarily attributed to agricultural, industrial, and wastewater runoff from the watershed to the receiving lakes (Akhtar, 2021; Bhateria & Jain 2016; Sharma et al., 2024). The pollutant release from these sources, when discharged to the lake without appropriate treatment, may cause an increase in toxicity and nutrients, leading to Harmful Algal Blooms (HABs; Carpenter et al., 1998; Smith, 1999). The blooms will have multidimensional consequences, including the disruption of aquatic biodiversity, anoxia, fish kill, and a serious risk to human health (Berdalet et al., 2016). The algal bloom issue has become a particular area of research topics, not only in developing regions. For instance, the water resources in North America and Europe are relatively well-managed, but the waterbodies in these regions are facing substantial water quality degradation because of climate change, climate variability, point and non-point source pollution (Elmadani, 2024; Minnesota Pollution Control Agency, 2020).

To effectively address these multifaceted challenges associated with water quality assessment, Multivariate statistical techniques were introduced and popularly used by scientists and researchers (Kazi et al., 2009; Kumar et al., 2008; Singh et al., 2004). Since traditional water quality assessments. may struggle to unravel the hidden patterns and interdependencies across multiple physicochemical and biological parameters (Kachroud et al., 2019; Kazi et al., 2009; Pesce & Wunderlin, 2004)*,* multivariate statistical approaches, especially Principal component analysis (PCA), simplify the complex datasets by converting them into interpretable patterns of datasets with uncorrelated components, pinpointing the main factors dominating the pollution source (Shrestha & Kazama, 2007; Singh et al., 2004)*.* PCA facilitates decision-making for lake management, enabling researchers to classify pollutants, monitor spatiotemporal dynamics, and trends (Hou, 2022). It aids in determining key environmental factors for deteriorating water health (Singh et al., 2004) and assessment of spatiotemporal trends (Dessie Tibebe, 2022). Therefore, it has been effectively utilized in diverse geographical settings across the world (Hou, 2022; Shrestha & Kazama, 2007; Singh et al., 2004).

Although the reduction in dimensionality is a valuable advantage of PCA (Hasan & Abdulazeez, 2021), its importance in environmental monitoring is beyond simplification. In environmental monitoring, datasets often include a wide array of interrelated parameters such as Dissolved Oxygen (DO), temperature, pH, turbidity, chlorophyll-a, and blue-green algae (BGA). By applying PCA, underlying structures can be identified within the datasets and isolated as the dominant factor of water health. Despite the proliferation of PCA-based studies, several notable gaps persist. Many investigations prioritize large-scale aquatic systems or rely on satellite-derived data, which may overlook localized dynamics and in situ variability (Dodangeh et al, 2025). Multivariate water quality analysis of the mid-sized reservoirs, such as Tappan Lake of Eastern Ohio, which serves as a key resource for flood control, recreational activities, and water supply, would be extremely beneficial for the restoration and HAB management in the lake. Even though the Tappan Lake supports a wide range of ecological and human activities, stakeholders have expressed serious concern as the lake has encountered Harmful Algal Blooms (HABs) in recent years. This alarming consequence of eutrophication is the emergence and proliferation of HABs, which can release toxins (Lan et al., 2024). This phenomenon has not only threatened the aquatic environment and ecosystem stability (Lan et al., 2024) but also recreational use and public health, leading to the need for a comprehensive and scientifically robust assessment of water quality. The frequent and intense HABs cause a serious impact on the aquatic environment and biodiversity in addition to endangering public health, ecological integrity, and recreational safety (Brenckman et al., 2025). This diminishes the lake's value and worth, underscoring the urgent need for a rigorous, data-driven diagnostic framework (U.S. EPA, 2025; Priya et al., 2023).

This study seeks to bridge that gap by applying PCA in a high-resolution, multi-temporal dataset from Tappan Lake and captures nuanced interactions among the variable parameters such as temperature, pH, dissolved oxygen (DO), turbidity, blue-green algae (BGA), and chlorophyll-a (Chl-a). By isolating the principal components that govern water quality degradation, this research aims to identify the pattern of dominant variables, characterize spatiotemporal patterns, and provide a framework to advocate for multivariate statistical tools in aquatic environment management. This will further help in a deeper understanding of the lake’s ecological status while demonstrating the broader application of PCA in reservoir management and freshwater management.

While PCA has been extensively implemented in various studies, this study applies repeated PCA across distinct lake zones and temporal periods using high-resolution vertical profiles to investigate localized spatiotemporal patterning. To the best of the author’s knowledge, this study is unique as it attempts to investigate the temporal and spatial water quality dynamics of the lake, including lake mixing stratification and lake overturn across the various time periods by employing several PCA models to identify how different water quality variables will be predominant to represent the lake water quality in various time periods. Even though direct nutrient loading was not quantified in this monitoring campaign, the study uses high-resolution physicochemical surrogates to hypothesize mechanistic patterns associated with eutrophication. The outcomes not only enhance Tappan Lakes’ ecological understanding but also deepen insight into its resilience and provide a robust framework for water quality assessment of mid-sized lakes/reservoirs.

### Study area

**Fig. 1:**
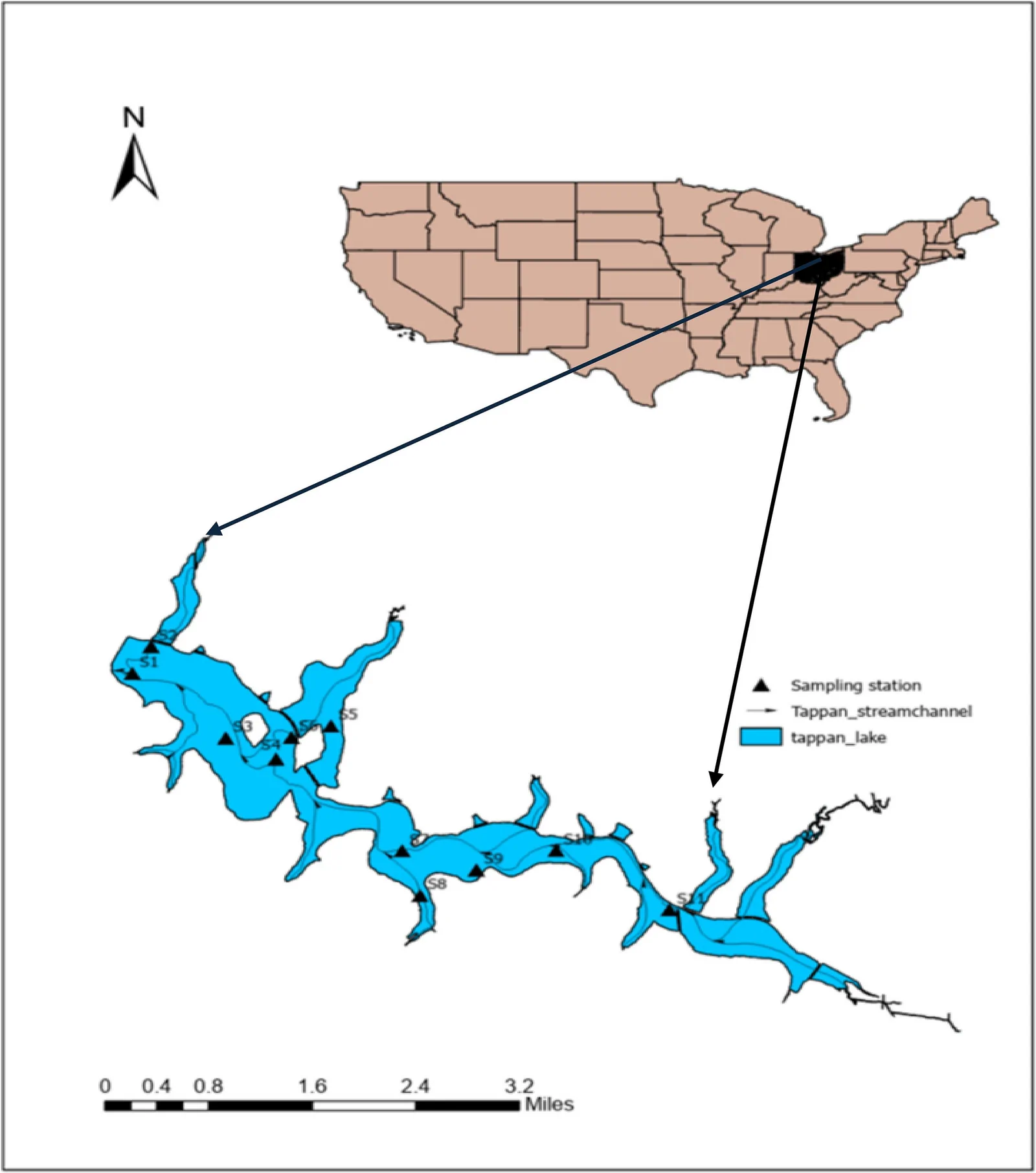
Study area: field monitoring stations (S1–S11) used for water quality modeling of Tappan Lake, Ohio, USA

This study was conducted in a freshwater reservoir, Tappan Lake (Fig. 1), which is in Harrison County of East-Central Ohio, USA (40.3597° N, 81.1848° W), within the Muskingum River Basin. The lake was initially constructed by the U.S. Army Corps of Engineers in 1936 for flood control. Currently, the Muskingum Watershed Conservancy District (MWCD) and the United States Army Corps of Engineers (USACE) jointly manage this reservoir. The approximate area covered by the lake is 9.5 km^2^ with a normal pool elevation of 274.1 m above sea level and a maximum depth of 10.4 m near the dam. Little Stillwater Creek, a major tributary of the Tuscarawas River, and numerous ephemeral tributaries are the major sources of inflow in this lake, contributing to its ecological balance and nutrient loading. The outlet of the lake is on the southeastern end of the reservoir, known as the Tappan dam, which drains Stillwater Creek and eventually discharges to the Muskingum River of the larger Ohio River Basin.

This lake was selected for research owing to its ecological significance and reported environmental challenges that are incurred due to excessive nutrient enrichment, exponential algal bloom, etc. The watershed is predominantly covered by forested land (84%), and a small part is covered by residential and agricultural areas (16%). The average annual precipitation of Tappan Lake is 1085 mm, and the mean air temperature ranges from 29 °C in summer to 1 °C in winter. Historical water quality data obtained from the U.S. Geological Survey (USGS Station 03128000) and the National Water Quality Portal (Site ID: 21OHIO_WQX-201931) revealed that stratification occurs from early spring and extends to early fall. Observation also reflects elevated chlorophyll-a concentrations, and persistent shifts in pH and BGA levels. Increments in these parameters are recognized as the prominent variables contributing to variance in eutrophic lake systems (U.S., 2020). These conditions position Tappan Lake as a representative lentic system for biochemical response to inter-annual climatic variability, watershed-derived nutrient and sediment inputs, and in-lake management interventions.

## Materials and methods

### Site selection

To accurately reflect the spatial heterogeneity of biological and physicochemical conditions within Tappan Lake, high-resolution water quality data were collected on a biweekly basis across 11 strategically selected sampling locations. These sites encompass diverse hydrological zones within the reservoir to rigorously characterize the spatiotemporal variability in Tappan Lake’s water quality. Sites included inflow zones, mid-lake zones, outlet regions, and both maximum and minimum depth locations to assess vertical and lateral variability in temperature, DO, pH, turbidity, chlorophyll-a, conductivity, and BGA. Sampling was conducted using a calibrated YSI EXO3s Sonde, generating a high-resolution vertical profile at every 1-m depth interval throughout the water column. For the PCA, the 1-m interval vertical profile data were depth-averaged per site-date to create a single representative value for the water column at each station. This standardization was necessary to perform consistent multivariate analyses across the entire reservoir, as sampling stations varied significantly in total depth. Furthermore, at many of the shallower stations, the water column remained relatively well-mixed throughout the study period, meaning the depth-averaged values closely approximated in situ conditions with minimal loss of detail. While this provides a robust horizontal comparison, we acknowledge that depth-averaging may mask specific hypolimnetic oxygen depletion dynamics, which are addressed separately via profile analysis.

### Data acquisition and preprocessing

Field monitoring campaigns were conducted biweekly from April 2024 through October 2025, spanning two consecutive open-water reservoir monitoring time periods. A calibrated YSI EXO3s Sonde, a xylem brand, was used to measure temporal, spatiotemporal, high-resolution water quality parameters, including DO (mg/L), lake water temperature (°C), pH, conductivity (µs/cm), turbidity (NTU), chlorophyll-a (µg/l), and BGA (µg/l) were measured at 11 sampling stations with observations recorded at every one meter depth interval. All measurements were collected by trained field personnel at biweekly intervals using the sampling approach suggested by the Ohio EPA on the same day within a 3-to-4-h time window spanning from the first to the final stations to minimize the temporal variability. The data were automatically recorded by the device in real time and retrieved later on the computer. Before analysis, the dataset underwent a structured preprocessing workflow to ensure consistency, completeness, and suitability for multivariate statistics. First, all raw profiles were visually inspected to identify sensor malfunctions, spurious spikes, and physically implausible values. Such values were flagged and removed when they could be clearly attributed to instrument noise or deployment artifacts. Second, missing values arising from occasional sensor dropouts were retained as NA and excluded listwise from PCA and correlation analyses, rather than imputed, to avoid introducing artificial structure into the dataset. Third, for each station-date combination, the 1 m interval vertical profile was depth-averaged to obtain a single representative value per parameter, as described in the Site Selection subsection. Finally, variables exhibiting extremely low variance or strong redundancy were screened but, in this study, all core physicochemical and biological parameters were retained for multivariate analysis.

To contextualize the thermal dynamics of the lake, daily average air temperature data were retrieved from the USGS Station 03128000 (Tappan Lake). This site-specific meteorological data provided a direct benchmark for evaluating the thermal coupling between ambient air and surface water temperatures across the sampling stations.

### Statistical analysis

In order to conduct the statistical analysis, at least these six steps were undertaken: (i) All the collected data were compiled properly in rows and columns, showing each measured variable, and the data were also reviewed for consistency; (ii) The data were screened to exclude the extreme outliers and evaluated for their consistency from one station to another; (iii) PCA was performed in XLSTAT using the correlation matrix, and the variables were centered with z-score normalization; (iv) The variance and eigenvalues after each PCA were examined; (v) Score plots were generated to examine spatial and temporal patterns of the dataset to interpret lake mixing, stratification, and temporal transitions; (vi) finally the scree plot generated by XLSTAT were visually evaluated to see the meaningful relationship and patterns. Multivariate analysis provided metrics such as mean, standard deviation, and range, which served as the foundation for our data sets. The range was crucial in finding anomalies in the data sets collected. It also showed where inconsistencies lay within the data sets. It also provided correlation statistics among the data points, highlighting which parameters have the greatest impact on the lake’s ecosystem and revealing how consistent these relationships are across different sites and seasons.

PCA was employed across multiple sampling locations and time periods around the lake to reduce dimensionality and emphasize the most influential variables. All multivariate analyses, including PCA, Pearson correlation analysis, and cluster analysis, were performed using XLSTAT 2025.1. The application of disaggregated PCA models across different seasons and lake zones yielded specific eigenvalues and factor loadings, which were instrumental in identifying significant groupings, correlations, and outliers within the data. The graphical representations further enhanced interpretability, allowing for a clearer visualization of variable importance and clustering behavior across sampling sites and time periods. A graphical representation called a scree plot was plotted to illustrate the proportion of variance explained by each principal component (F1 through F11). This plot played a crucial role in identifying the components that accounted for the most significant variability within the dataset. In all PCA analyses conducted, the first two principal components (F1 through F2) consistently emerged as the most crucial to explain the total variance. PCA has the ability to synthesize complex datasets into meaningful visualizations, such as the scree plot, which allows for efficient identification of key patterns. This enabled the research to focus analytical efforts on the most informative variables.

PCA was initially performed on each individual sampling date and station to assess high-resolution variability. From this comprehensive analysis, representative PCA models were selected for Table 2a and b to characterize the dominant drivers of the reservoir zones and temporal windows. These representative models were chosen based on their ability to capture the median variance and loading patterns identified in the broader dataset, providing a concise yet statistically robust overview of the system's multidimensional behavior. The geographical scope allowed for an assessment of spatial variability within the lake, while the use of multivariate analysis provided insight into underlying patterns within the datasets through PCA. Furthermore, combining conclusions from other datasets will help support the trends seen in the datasets collected.

Agglomerative Hierarchical Clustering (AHC) was performed on the depth-averaged dataset of sampling events to objectively classify water quality regimes. The analysis utilized Ward’s Method as the linkage criterion and Euclidean Distance as the dissimilarity metric to determine the relationship between monitoring stations and sampling dates. This approach provides a robust, data-driven validation of the spatial zonation (Inlet, Mid-lake, and Outlet) and identifies temporal shifts occurring during thermal stratification.

To rigorously quantify the drivers of environmental heterogeneity, a one-way Permutational Multivariate Analysis of Variance (PERMANOVA) was executed using the vegan package in R (v4.3.3) via the Posit Cloud environment. The analysis was performed on a Euclidean distance matrix derived from z-score normalized variables to ensure equal weighting of parameters with varying units. Statistical significance was assessed through the adonis2 function using 999 Monte Carlo permutations (*α* = 0.05). This non-parametric approach allowed for the partitioning of total multivariate variance (*R*^2^) and provided statistical validation of the spatial (zonal) and temporal gradients previously identified via PCA. By utilizing permutation-based testing, the analysis provided a robust evaluation of the reservoir's limnological structure without the strict requirements of multivariate normality.

## Results and discussion

### Raw data trends

**Fig. 2:**
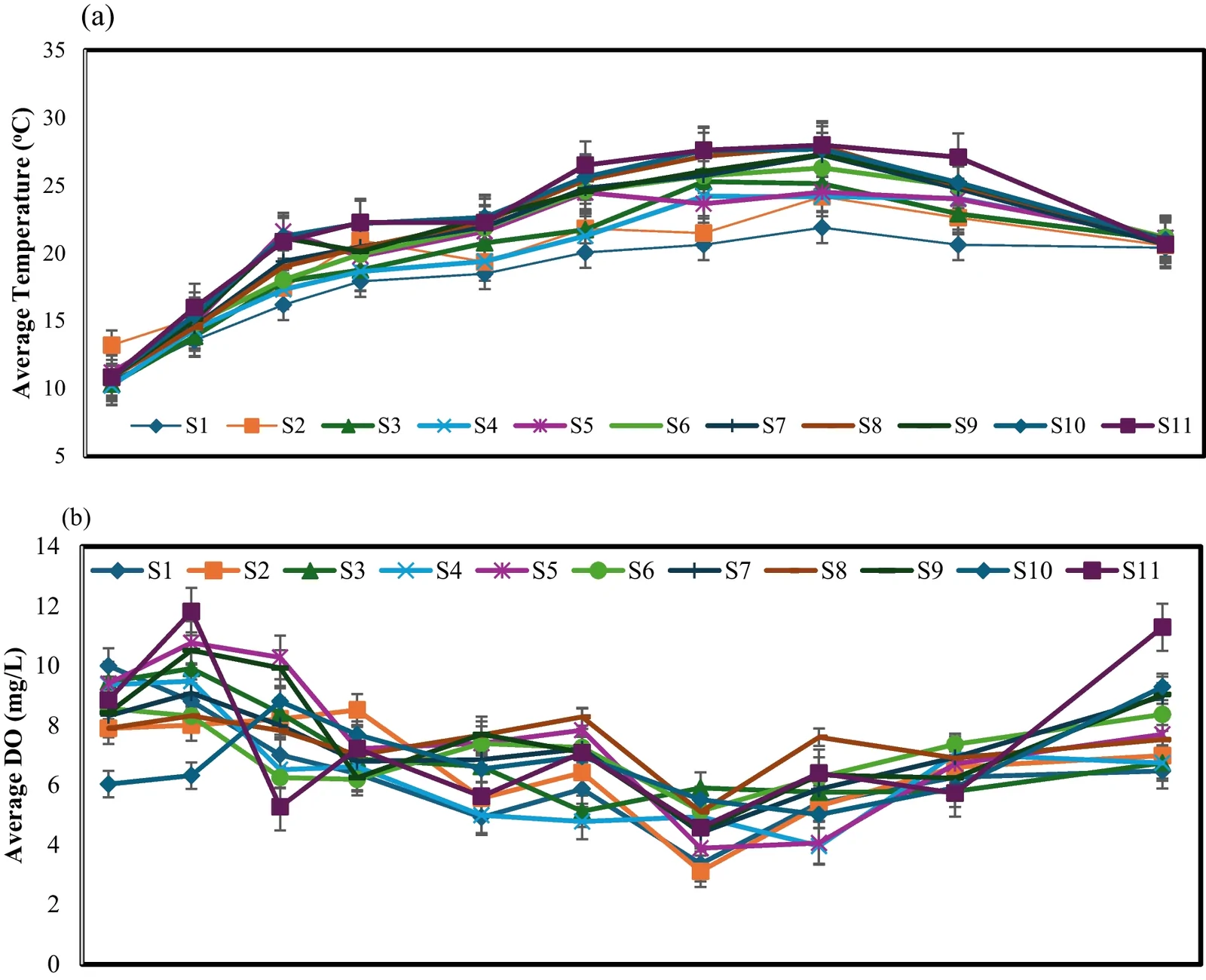

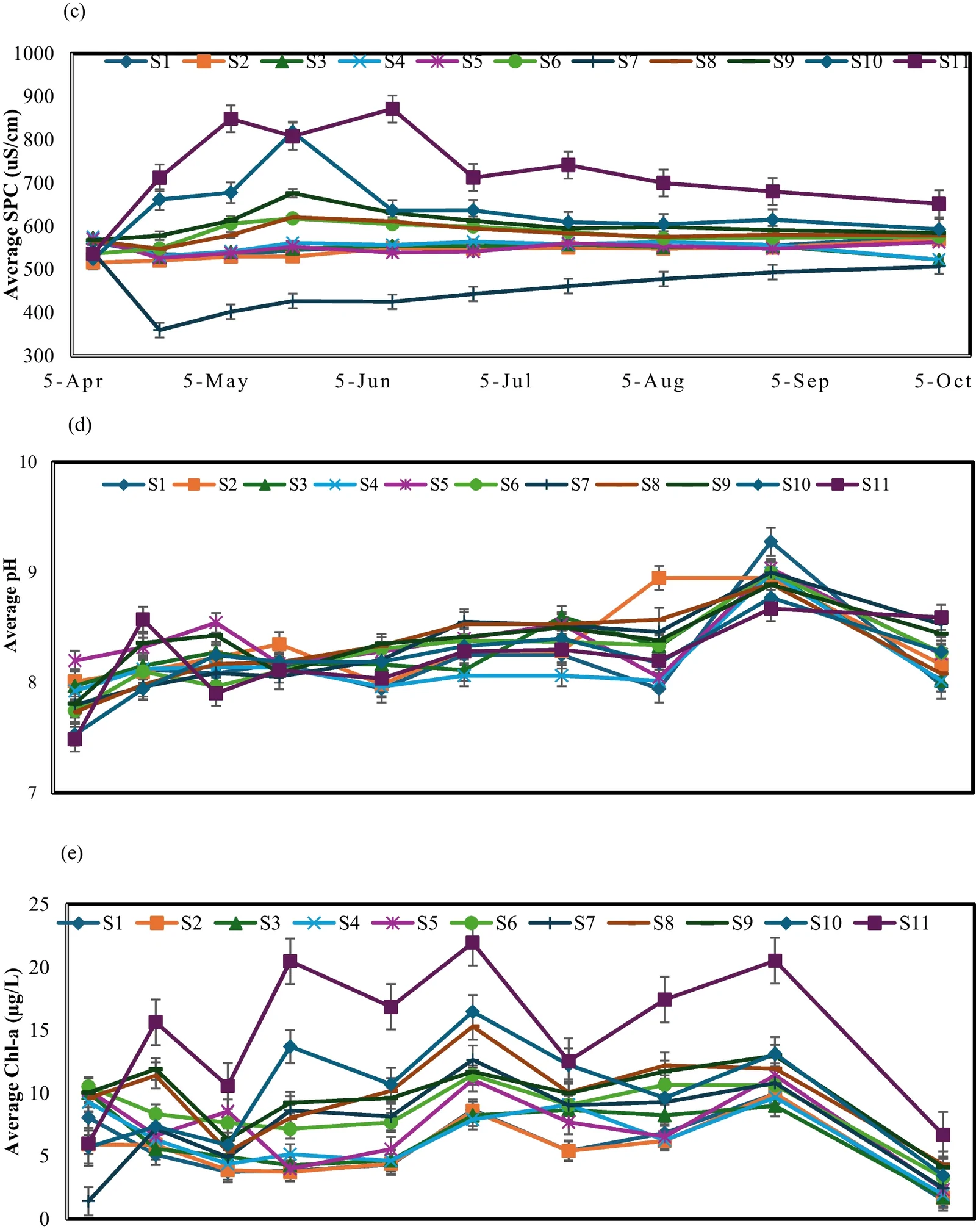

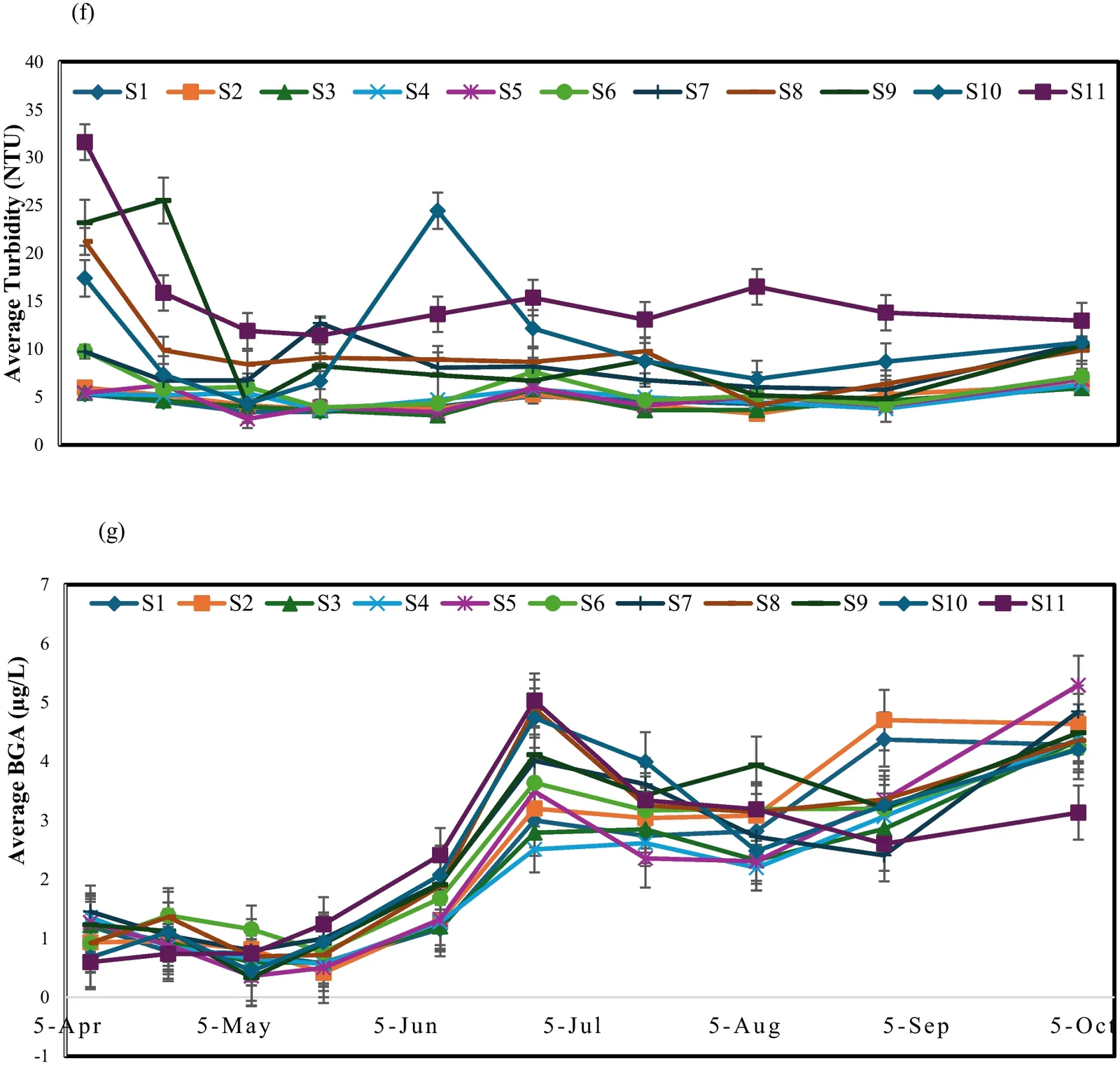
The trend of some water quality parameters across sampling stations (S1-S11), including **a** temperature, **b** DO, **c** specific conductivity, **d** pH, **e** Chl-a, **f** Turbidity, and **g** Blue-green algae. Error bars represent ± 1 standard deviation

Raw water quality of Tappan Lake exhibited a clear and physically consistent structure that aligned well with subsequent PCA. To illustrate how variability manifests both across space and through time, we constructed a representative summary table (Table 1). In this table, the “Spatial” statistics describe the minimum, maximum, mean, and standard deviation of depth-averaged parameters across all 11 stations for a single sampling date (April 23, 2024), whereas the “Temporal” statistics describe the same metrics across all sampling dates at a single representative station (S6). This dual structure is intended to distinguish horizontal (spatial) heterogeneity from temporal variability at a fixed location. Throughout the year, the lake exhibited well-defined temporal patterns in thermal and oxygen dynamics (Li, 2021; Wetzel, 2001). The temporal patterns shown in Fig. 2a reflect expected limnological behavior, but their value lies in documenting how these processes manifest specifically in Tappan Lake during the monitoring window. Although the general increase in temperature from spring to summer and the decline toward fall is well-established in lake ecology, the magnitude, timing, and spatial heterogeneity of these transitions differ among reservoirs depending on watershed inputs, morphometry, and hydrological residence time. Average water temperatures across the column soared from approximately 10–14 °C (early spring) to 20–26 °C (mid-summer), followed by a decline during fall (15–18 °C) as shown in Fig. 2a. Because of shallower bathymetry and enhanced surface runoff exposure, inlet zones (e.g., S10-S11) experienced warmer water (Thornton & K, B, 1990) than those of the higher depth outlet zones (e.g., S1-S2). On the other hand, this warming trend was inversely mirrored by the DO concentration (Wetzel, 2001), being highest in spring (> 8 mg/L), which decreased sharply during the summer (< 2 mg/L). This is not surprising, as water temperature increases, the solubility of dissolved oxygen decreases due to the high molecular motion of the gases. When temperature rises during the late spring or early summer, generally DO decreases even if the oxygen production is constant, mainly due to the thermal stratification that prohibits vertical mixing (Ma, 2025; Smith & Bella, 1976).

**Table 1:** Representative summary statistics of depth-averaged water quality parameters illustrating spatial and temporal variability in Tappan Lake. The “Spatial” columns summarize variability across all 11 stations for a single representative sampling date (April 23, 2024), while the “Temporal” columns summarize variability across all sampling dates at a single representative station (Station 6)

Variables	Spatial				Temporal			
	Minimum	Maximum	Mean	Std. deviation	Minimum	Maximum	Mean	Std. deviation
Temperature	13.20	15.99	14.57	0.81	10.89	26.30	20.82	5.05
DO	6.32	11.82	9.21	1.53	5.13	8.57	7.11	1.13
SPC	359.48	712.10	549.51	88.63	536.53	618.16	582.01	26.13
pH	7.94	8.58	8.15	0.20	7.75	9.00	8.26	0.33
Turbidity	4.51	25.51	8.88	6.39	3.91	9.79	5.87	1.87
BGA	0.74	1.39	1.01	0.22	0.76	4.23	2.33	1.28
Chl-a	5.12	15.65	8.31	3.30	3.30	11.45	8.65	2.42

DO underwent a recovery phase in the fall, returning to levels similar to the initial spring observations (Fig. 2b). The autumnal rise in dissolved oxygen (DO) across sampling sites reflects the onset of fall turnover, where the breakdown of thermal stratification facilitates vertical convective mixing and the subsequent re-oxygenation of the hypolimnion. Although having certain short-term spikes in the inlet zones suggesting conditions of nutrient enrichment and ion-rich runoff due to road salt input (Diaz, 2008; Ellen Foley, 2023; Kaushal et al., 2018), specific conductivity remained relatively constant (530–570 µS/cm). In contrast, S7 exhibited lower conductivity values (< 500 µS/cm) throughout the year (Fig. 2c). Even though the typical range of pH was from 7.9 to 8.3, the pH value of the lake was found to be > 9.0 during late summer (Fig. 2d), which was generally associated with the intense algal proliferation remaining neutral to slightly alkaline (Fig. 2e). The observed increase in pH is attributed to the rapid consumption of dissolved CO_2_ by phytoplankton and cyanobacteria during photosynthesis. This biological uptake shifts the carbonate equilibrium by reducing the concentration of carbonic acid (H_2_CO_3_), leading to a relative increase in carbonate and hydroxyl ions, which drives the water toward alkaline conditions (Zerveas et al., 2021). Influenced by sediment entrainment and watershed input by spring runoff, turbidity (Fig. 2f) was higher in the spring months (> 10 NTU) at inlet stations, whereas most of the stations recorded turbidity in the range of 3–6 NTU. BGA concentration was minimal during the spring months (< 1.5 µg/L) but spiked during the summer (> 3.0 µg/L), peaking at the inflow sites (Fig. 2g), showing nutrient-driven algal bloom hotspots. This is not surprising because the increased sediment concentration due to spring runoff inhibits cyanobacterial growth (Gu et al., 2022; Yang et al., 2022). In addition, the spring temperature is not suitable for cyanobacteria growth, as their growth is supported by relatively warmer temperatures during the summer (Huisman et al., 2018). Also, the strong vertical mixing due to spring lake overturn reduces the growth of cyanobacteria (Posch et al., 2012).

Notably, precipitation-driven inflows during the fertilizer transport substantial nitrogen and phosphorus from agricultural lands, accelerating algal bloom formation and compromising water quality at critical inflow sites (Ohio State University Extension, 2024; Ohio, Purdue, and University of Illinois, 2020).

A distinct bimodal temporal distribution was displayed by Chl-a, characterized by initial elevation in early spring, driven by nutrient enrichment from spring runoff (Newell, 2024; Steinman, 2015).

Spatially, outflow stations (e.g., S1 and S2) had stable pH, lower turbidity, and moderate Chl-a, typically exhibiting the characteristics of oxygen-rich, well-mixed zones maintained by continuous flow.

**Fig. 3:**
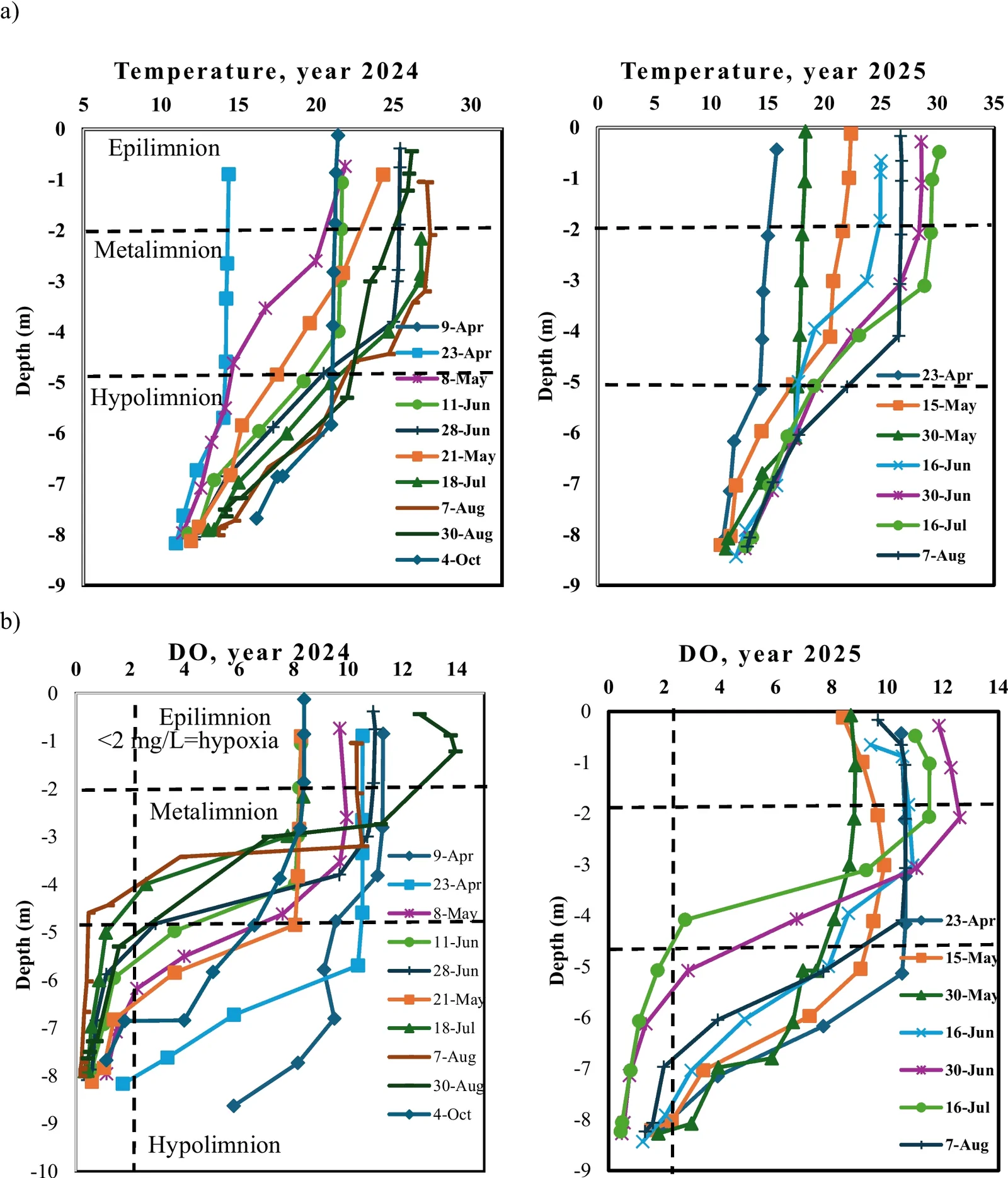
**a** Water temperature. **b** DO profile for exemplary site S1 (outlet) representing temporal stratification of the lake for the years 2024 and 2025

Mid-lake stations (e.g., S5, S6, S7) are influenced by thermal stratification, which led to temporary hypolimnetic oxygen depletion (Figure 3) and moderate algal biomass under a mixing cycle (Ling, 2019). In contrast, inflow stations (e.g., S11) displayed a significant increase in turbidity, BGA, Chl-a, and pH (>9) during summer. The increased Chl-a and BGA during the summer was supported by nutrient enrichment due to internal loading (Nürnberg, 1984), shallower depth, and stagnant hydrodynamics (Reynolds, 1994). Overall, the data analysis portrayed a clear and systematic shift from spring equilibrium to summer eutrophic state with a partial sign of restoration in early fall, which is consistent with the findings suggested by Stockwell and Borowski (2008).

By mid‑May to late June, a thermocline developed between 3–5 m depth, with surface temperatures rising to 20–25 °C and DO declining below 4 mg L⁻1 beneath the thermocline. Peak stratification occurred in July–August, when hypoxic conditions (< 2 mg L⁻1) extended below 5 m. The October profile revealed re‑oxygenation and temperature homogenization, consistent with fall turnover. These representative profiles, collected on discrete dates within a single year, capture the intra‑annual transitions in Tappan Lake rather than multi‑year or continuous temporal cycles, clarifying the lake’s dynamic thermal and oxygen structure.”

### Principal component analysis (PCA)

The eigenvalue summary for spatial and temporal principal components in Tappan Lake water quality has been reported in Table 2.

**Table 2:** Eigenvalue Summary for a) Spatial and b) Temporal Principal Components in Tappan Lake Water Quality

**a**									
	**Outlet zone**			**Mid-lake zone**			**Inlet zone**		
	**F1**	**F2**	**F3**	**F1**	**F2**	**F3**	**F1**	**F2**	**F3**
**Eigenvalue**	2.739	1.916	1.561	3.250	1.642	1.101	3.497	1.485	1.123
**Variability (%)**	39.127	27.374	22.293	46.427	23.452	15.722	49.964	21.214	16.045
**Cumulative %**	39.127	66.501	88.794	46.427	69.879	85.601	49.964	71.178	87.222
**b**									
	**Spring**			**Summer**			**Fall**		
	**F1**	**F2**	**F3**	**F1**	**F2**	**F3**	**F1**	**F2**	**F3**
**Eigenvalue**	4.437	1.216	0.587	3.250	1.642	1.101	3.902	1.628	0.872
**Variability (%)**	63.389	17.377	8.381	46.427	23.452	15.722	55.740	23.258	12.455
**Cumulative %**	63.389	80.765	89.146	46.427	69.879	85.601	55.740	78.998	91.453

F1, F2, and F3 represent the first three principal components, capturing the dominant gradients of variability in water quality

**Fig. 4:**
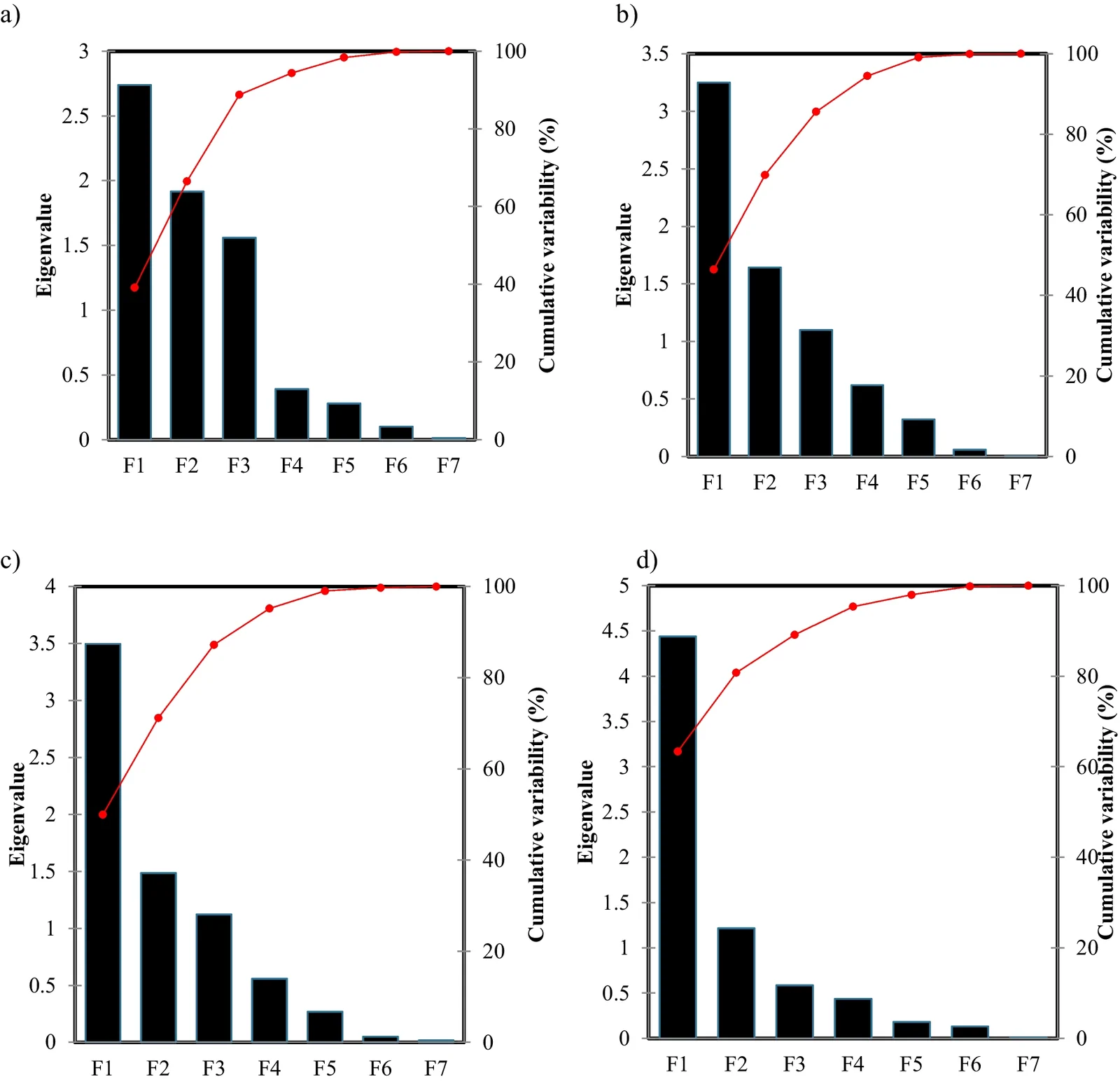

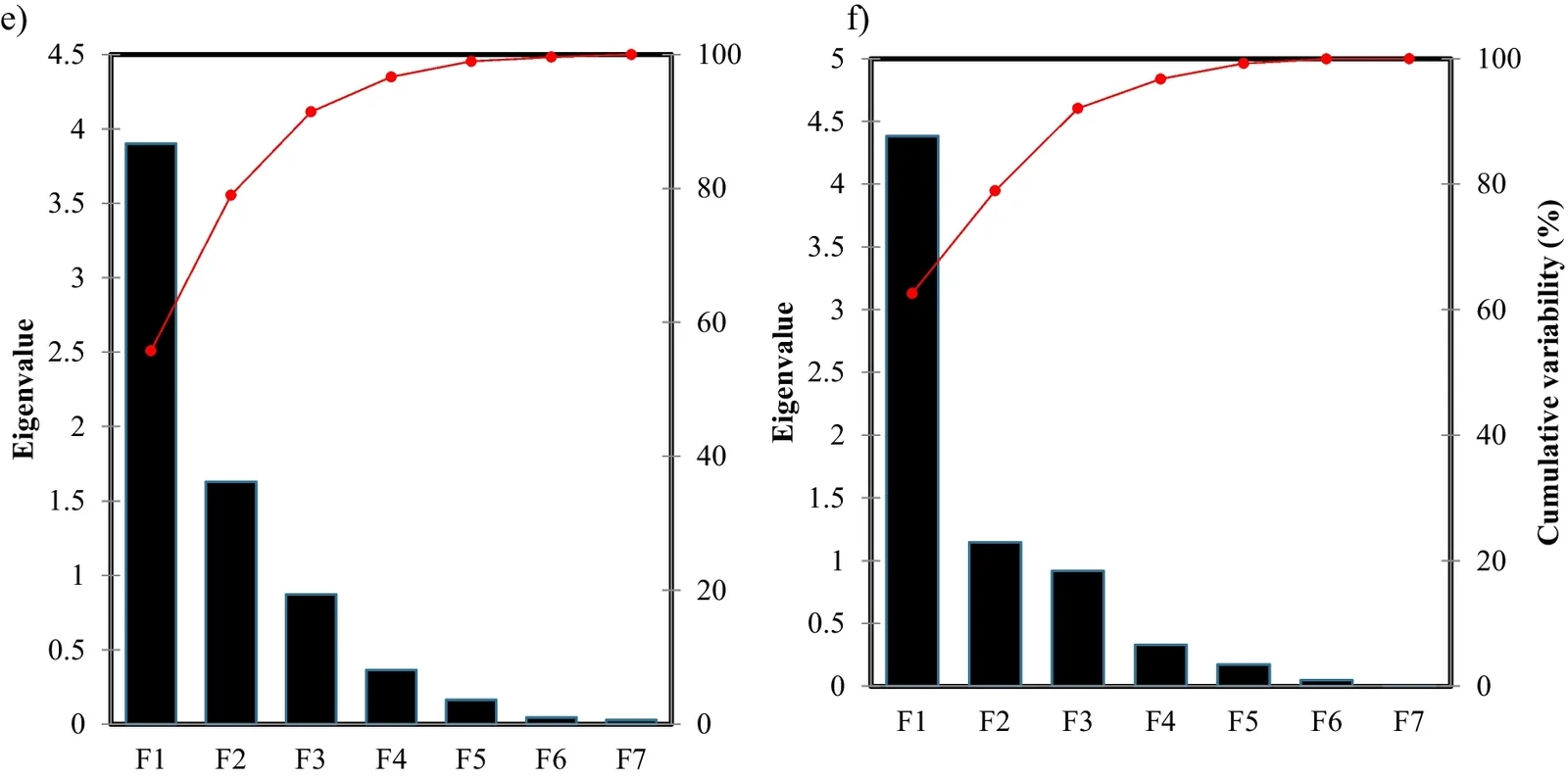
Scree plot:—Spatial: **a** outlet zone, **b** mid-lake zone, **c** inlet zone; Temporal: **d** spring, **e** summer, **f** fall

**Fig. 5:**
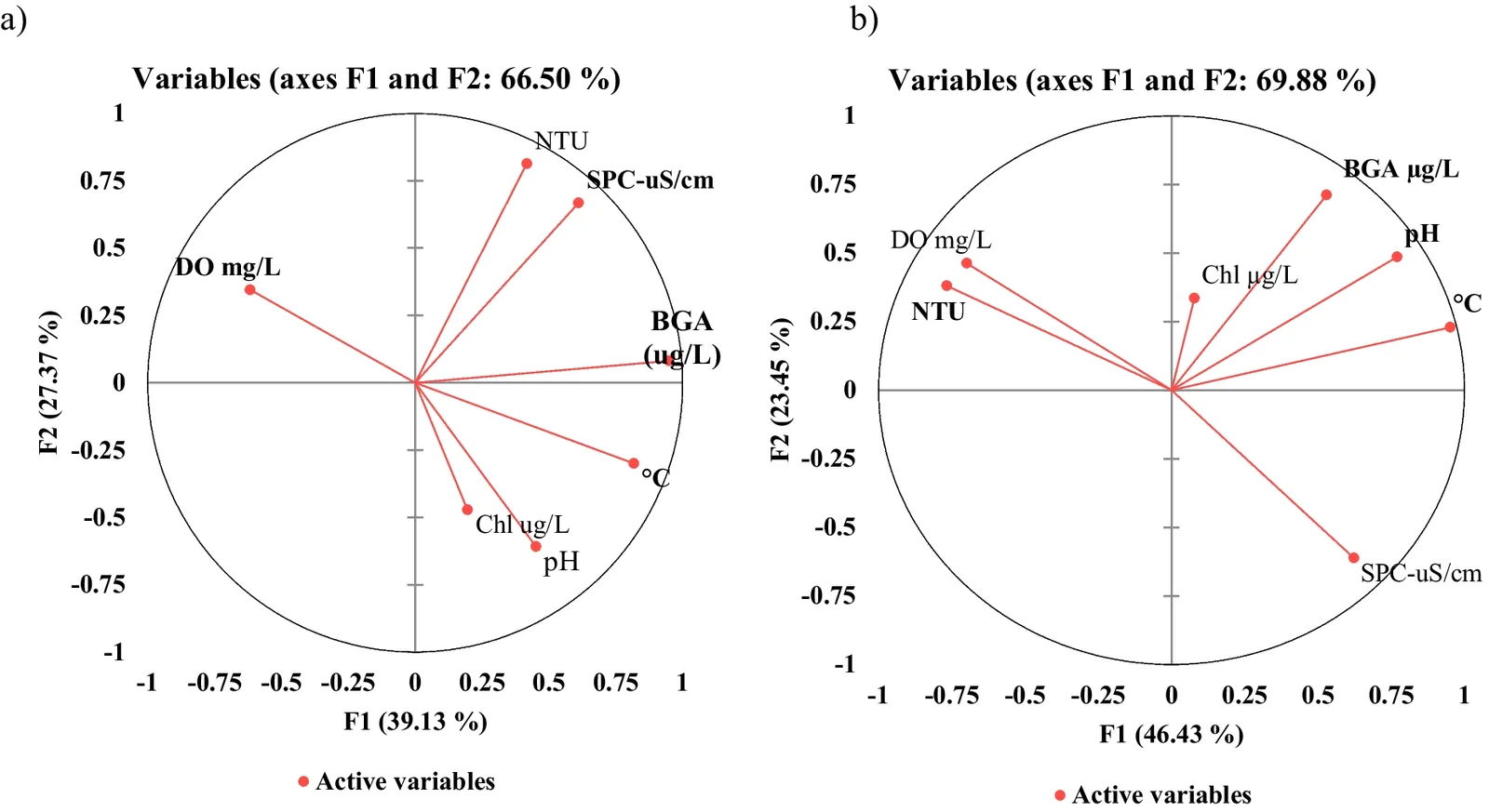

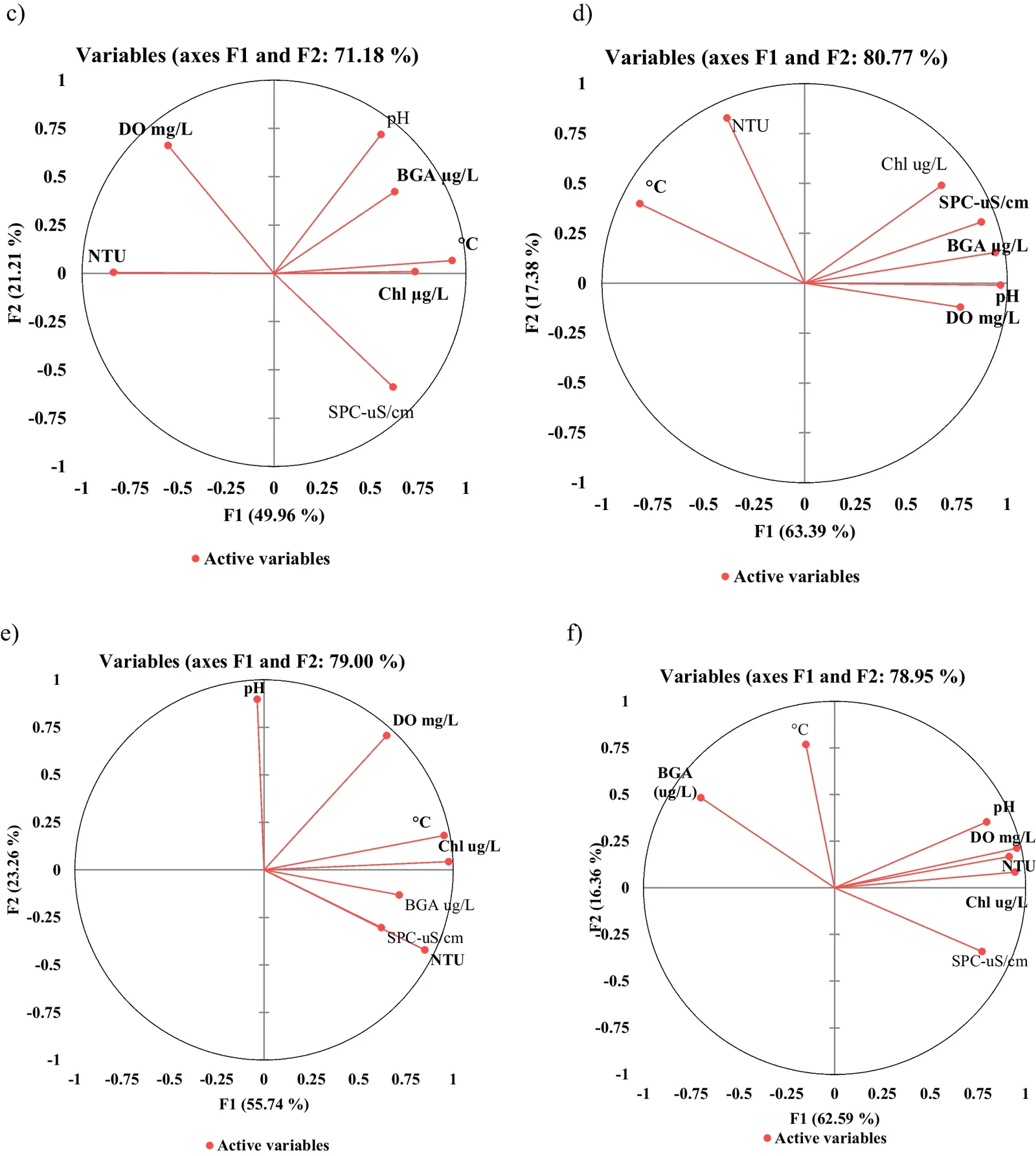
Multivariate analysis of water quality parameters of Tappan Lake using PCA to extract the Principal Components:—Spatial: **a** outlet zone, **b** mid-lake zone, **c** inlet zone; Temporal: **d** spring, **e** summer, **f** fall

Principal Component Analysis (PCA) was performed on the depth-averaged dataset to identify dominant gradients in water quality parameters. The first three components (F1–F3) collectively explained ≈ 82% of the total variance, with F1 and F2 accounting for ≈ 70%. The Principal Component Analysis (PCA) revealed two dominant gradients governing approximately 70% (Fig. 4) of the total variance of water quality dynamics of Tappan Lake (Fig. 5). The first principal component (F1) demonstrated the spatiotemporal structure of the thermal-biogeochemical axis governed by positive loading of temperature, pH, Chl-a, and BGA, contrasted by strong negative loading of DO (Fig. 5). This suggested that warming and increased photosynthetic activity covary with DO depletion and higher HAB (Hou, 2022; Shrestha & Kazama, 2007; Smith, 1999). These patterns reflect the response of phytoplankton biomass to unmeasured nutrient loading and water residence time, which is consistent with Wetzel (2024). F2 expressed biological and sedimentary influences, with positive loadings of BGA and Turbidity, and a negative loading of DO (Fig. 5), indicating oxygen depletion coinciding with bloom events and nutrient-rich inflows (Peng et al., 2021). Although F3 contributed additional variance (~ 12%), its loadings were diffuse and did not reveal a distinct ecological axis. Therefore, F1–F2 were retained for interpretation and visualization, as they effectively summarize the major ecological processes, thermal stratification and eutrophication without overcomplicating the biplot. Spatially, the outlet stations, such as S1 and S2, exhibited a stable structure primarily dominated by temperature and BGA with minor bloom episodes (Table 3). On the contrary, mid-lake zones exhibited a strong bloom-hypoxic condition under summer stratification, creating an elevated risk of fish kill, when the DO level dropped below 3–4 mg/L, which was supported by various past studies (U.S. EPA, 2025; Diaz, 2008). In the meantime, the data from inlet stations suggested bloom conditions because of the highest SPC and turbidity. This confirmed the nutrient enrichment and road salt input in the lake from the watershed due to increased conductivity and turbidity as reported in various studies (Dessie Tibebe, 2022; Ellen Foley, 2023; Kaushal et al., 2018). While elevated SPC in the inlet stations is consistent with watershed inputs such as road salt (Kaushal et al., 2018), it remains an indicator of general ionic strength rather than a direct diagnostic of chloride without further ion chemistry.

**Table 3:** a) Spatial and b) temporal PCA factor loadings identifying dominant variables across zones and temporal periods

a)									
	**Outlet zone**			**Mid-lake Zone**			**Inlet Zone**		
**Parameters**	**F1**	**F2**	**F3**	**F1**	**F2**	**F3**	**F1**	**F2**	**F3**
**°C**	0.818	−0.299	−0.443	0.952	0.229	−0.012	0.928	0.066	0.291
**DO **	−0.617	0.345	0.647	−0.699	0.463	−0.326	−0.552	0.662	−0.427
**SPC**	0.611	0.668	0.099	0.623	−0.612	−0.148	0.620	−0.589	−0.460
**pH**	0.451	−0.608	0.515	0.770	0.486	−0.016	0.558	0.719	−0.332
**NTU**	0.417	0.814	0.273	−0.767	0.381	0.005	−0.836	0.005	0.491
**BGA **	0.949	0.081	0.221	0.529	0.712	−0.339	0.629	0.423	0.540
**Chl-a**	0.196	−0.472	0.740	0.078	0.335	0.926	0.735	0.009	0.048
b)									
	**Spring**			**Summer**			**Fall**		
**Parameters**	**F1**	**F2**	**F3**	**F1**	**F2**	**F3**	**F1**	**F2**	**F3**
**°C**	−0.814	0.398	0.203	0.952	0.181	−0.098	−0.150	0.768	−0.620
**DO **	0.769	−0.120	−0.345	0.649	0.707	−0.024	0.954	0.212	0.131
**SPC**	0.872	0.307	−0.110	0.620	−0.303	0.685	0.772	−0.342	−0.311
**pH**	0.967	−0.010	0.084	−0.036	0.898	0.226	0.797	0.353	0.441
**NTU**	−0.383	0.828	−0.394	0.851	−0.421	0.090	0.915	0.168	0.175
**BGA **	0.942	0.154	−0.046	0.715	−0.132	−0.576	−0.700	0.483	0.367
**Chl-a **	0.675	0.490	0.500	0.977	0.043	0.028	0.944	0.084	−0.246

The spring season (Table 3) experienced a well-mixed, oxygenated regime with spring runoff-driven turbidity (Pesce & Wunderlin 2004) along with the road salt input, which elevated the ionic concentration (Ellen Foley, 2023; Kaushal et al., 2018). As the temperature increased, a pronounced thermocline developed, leading to strong summer stratification accompanied by elevated pH, intensified cyanobacterial biomass, and progressive DO depletion. Warmer surface waters combined with nutrient-rich inflows stimulated high photosynthetic activity, which raised pH and accelerated bloom proliferation (Lin et al., 2021). Subsequent decay of cyanobacterial biomass enhanced oxygen consumption in deeper layers, contributing to mid-summer hypoxia (Conover et al., 2016). Finally, a lake recovery with increased DO was observed after the disruption of stratification (U.S. EPA, 2020). These lake-specific patterns demonstrate that temperature, pH, and BGA are the dominant ecological drivers of variance in Tappan Lake, and that stratification strength, bathymetry, and nutrient-rich inflows jointly regulate the timing and severity of eutrophication dynamics.

### Pearson correlation analysis

The primary objective of this study was to evaluate the water quality of Tappan Lake by identifying the key physicochemical variables defining its variability patterns and elucidating their interactions and influence on algal growth dynamics. Distinct spatiotemporal trends were detected throughout monitoring sites, reflecting the interrelationship among the key physicochemical and biological parameters (Table 4). Because Pearson correlation quantifies relationships between variables rather than between stations, all correlation coefficients reported in Table 4 represent lake‑wide associations across the full dataset. Accordingly, the positive correlation between specific conductivity and turbidity (*r* = 0.717) reflects that higher ionic strength generally co‑occurred with elevated suspended solids across all samples, consistent with mineral‑rich or sediment‑laden inflows (Diaz, 2008; Ellen Foley, 2023; Kaushal et al., 2018). Correspondingly, temperature and BGA (*r* = 0.678) indicated warmer inflow and water stagnancy (Reynolds, 1994) were favoring BGA proliferation. These correlations describe variable-level relationships across the entire dataset, not station-specific pairings.

**Table 4:** Pearson’s correlation coefficients (exemplary) for investigated water quality parameters. (a) Spatial variation across sampling stations; (b) temporal variation across sampling dates. Bold values indicate statistical significance at the *α* = 0.05 level

(a)							
**Variables**	**°C**	**DO mg/L**	**SPC **	**pH**	**NTU**	**BGA **	**Chl-a**
**°C**	**1**	**−0.857**	0.208	0.283	−0.007	**0.678**	−0.011
**DO **		**1**	−0.110	−0.117	0.180	−0.369	0.116
**SPC **			**1**	−0.037	**0.717**	0.596	−0.122
**pH**				**1**	−0.207	0.500	0.593
**NTU**					**1**	0.539	−0.068
**BGA **						**1**	0.270
**Chl-a**							**1**
(b)							
**Variables**	**°C**	**DO **	**SPC**	**pH**	**NTU**	**BGA **	**Chl-a**
**°C**	**1**	**0.753**	0.434	0.068	**0.721**	**0.648**	**0.951**
**DO **		**1**	0.168	0.512	0.205	0.333	**0.684**
**SPC **			**1**	−0.115	**0.685**	0.162	0.589
**pH**				**1**	−0.318	−0.171	−0.038
**NTU**					**1**	**0.617**	**0.789**
**BGA **						**1**	**0.625**
**Chl-a **							**1**

Because the monitoring program generated multiple correlation matrices across different dates and stations, including every table in the main manuscript would be overwhelming and hinder readability. To maintain clarity, we present only a representative correlation table (Table 4) that illustrates the dominant variable-to-variable relationships observed across the dataset.

A notable correlation between pH and Chl-a (*r* = 0.593) suggested the elevation of the pH value during the photosynthetic process (Zerveas et al., 2021), whereas weak water temperature and DO correlation (*r* = 0.263) suggested the local mixing inside the lake due to turbulence and inflows, which is consistent with the previous study (Smith & Bella, 1976).

Mid-lake stations (S3-S9) showed somewhat similar patterns, where DO and temperature showed a moderately positive correlation (*r* = −0.479) consistent with water temperature stratification. Equally, a strong correlation between SPC and BGA (*r* = 0.810) represented the nutrient-driven algal proliferation (Nowicka-Krawczyk et al., 2022; Son, 2024); Chl-a and pH had a notable correlation (*r* = 0.563), which suggested the chlorophyll-induced alkalinity (Zerveas et al., 2021) in the lake. Comparison with stations at outlet zones, such as S10-S11, showed distinct differences with a strong negative correlation between DO and temperature (*r* = −0.857), indicating a classical relationship between DO and temperature. This suggests a classical thermal-oxygen relationship (Wetzel, 2001), where water-oxygen is reduced with an increase in water temperature. These patterns can be attributed to thermal stratification and biological oxygen demand, which potentially increase the risk of hypoxia (Deemer, 2025; Dugener, 2023), particularly in downstream habitats during summer months (Table 3).

Temporally, during the spring season, the lake exhibited a strong correlation of temperature with DO (*r* = 0.753) and Chl-a (*r* = 0.951), suggesting the occurrence of active mixing and photosynthesis (Kuo, 2024; Wetzel, 2001). A strong correlation between sediment-driven nutrient enrichment, i.e., Turbidity and Chl-a (*r* = 0.789), indicated the early bloom initiation. During summer, lakes exhibited characteristics of eutrophic lakes (Jane, 2021) with thermal stratification showing a strong negative correlation between temperature and DO (*r* = −0.604). This indicated the condition of hypoxia, where oxygen depletion (Zhang et al., 2015) occurs as water temperature rises. Concurrently, elevated BGA concentrations indicated increased cyanobacterial abundance, while a strong positive correlation between pH and BGA (*r* = 0.941) suggests that photosynthetic carbon uptake by algal biomass contributed to increased pH levels in the water (Zerveas et al., 2021). As a result, DO concentration fell below the 5 mg/L threshold, which is considered severely low for aquatic life (Dugener, 2023; Jenny et al., 2016) and may even approach the hypoxic threshold of < 2–3 mg/L under severe conditions. Followed by a fall, the system showed signs of recovery, including the disruption of stratification (Wang et al., 2023). Finally, the lake demonstrated a clear rebound from summer hypoxia towards improved oxygenation and stability (Fernández Castro, 2021; Wetzel, 2001) in the fall.

**Fig. 6:**
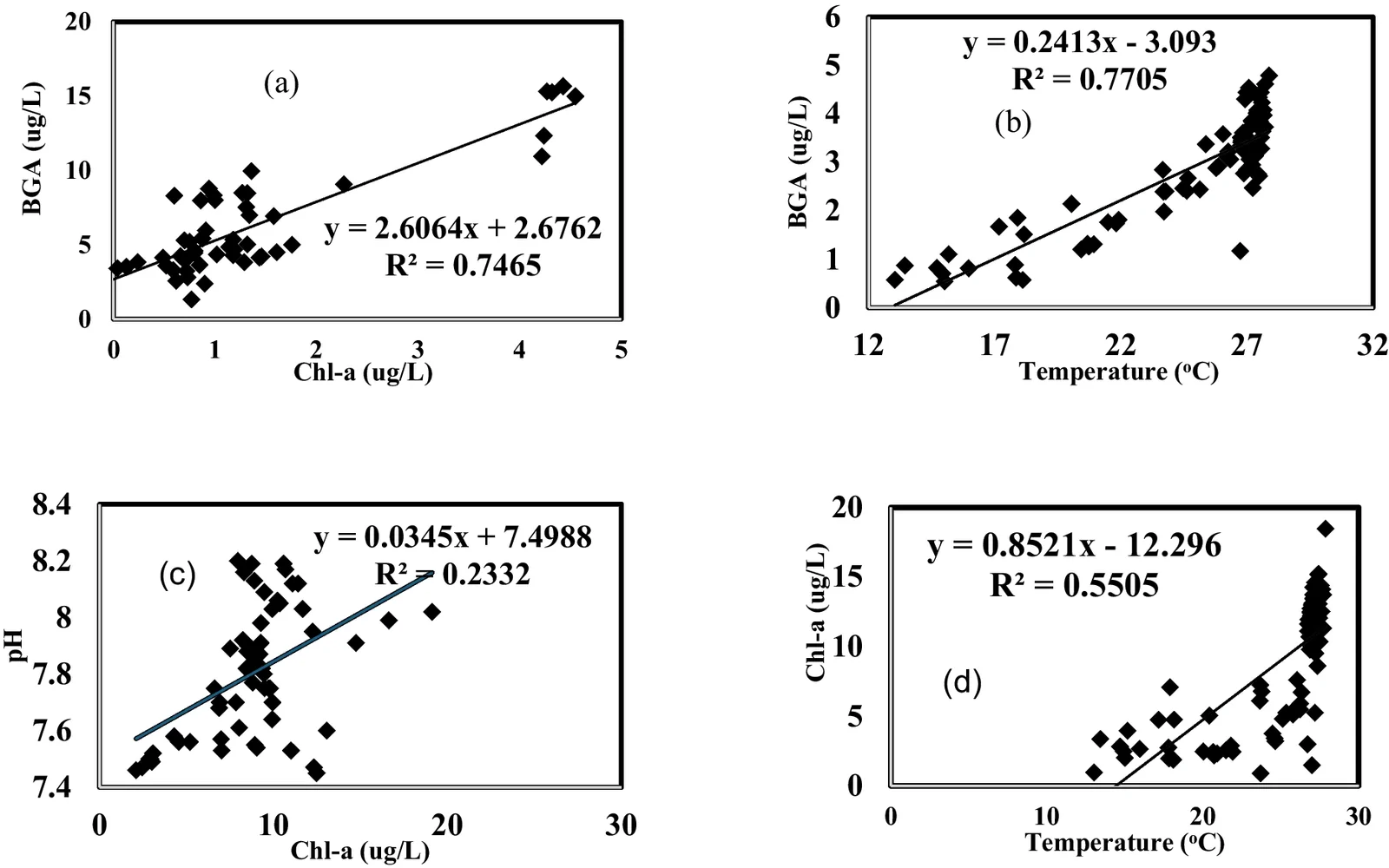
Correlation between **a** Blue-green algae (BGA) and chlorophyll-a (Chl-a), **b** BGA and temperature, **c** Chl-a and pH, **d** Chl-a and temperature

Figure 6 presents the bivariate correlations between key water quality parameters. A positive correlation between BGA and Chl-a (Fig. 6a) confirms the expected alignment of algal biomass indicators. The relationship between BGA and temperature (Fig. 6b) suggests a thermal influence on cyanobacteria dynamics, though the spread of data indicates that temperature is likely one of several contributing factors rather than a sole driver. In contrast, the correlations of Chl-a with pH (Fig. 6c) and temperature (Fig. 6d) exhibit weak linear trends and low R^2^ values.

### Cluster analysis

**Fig. 7:**
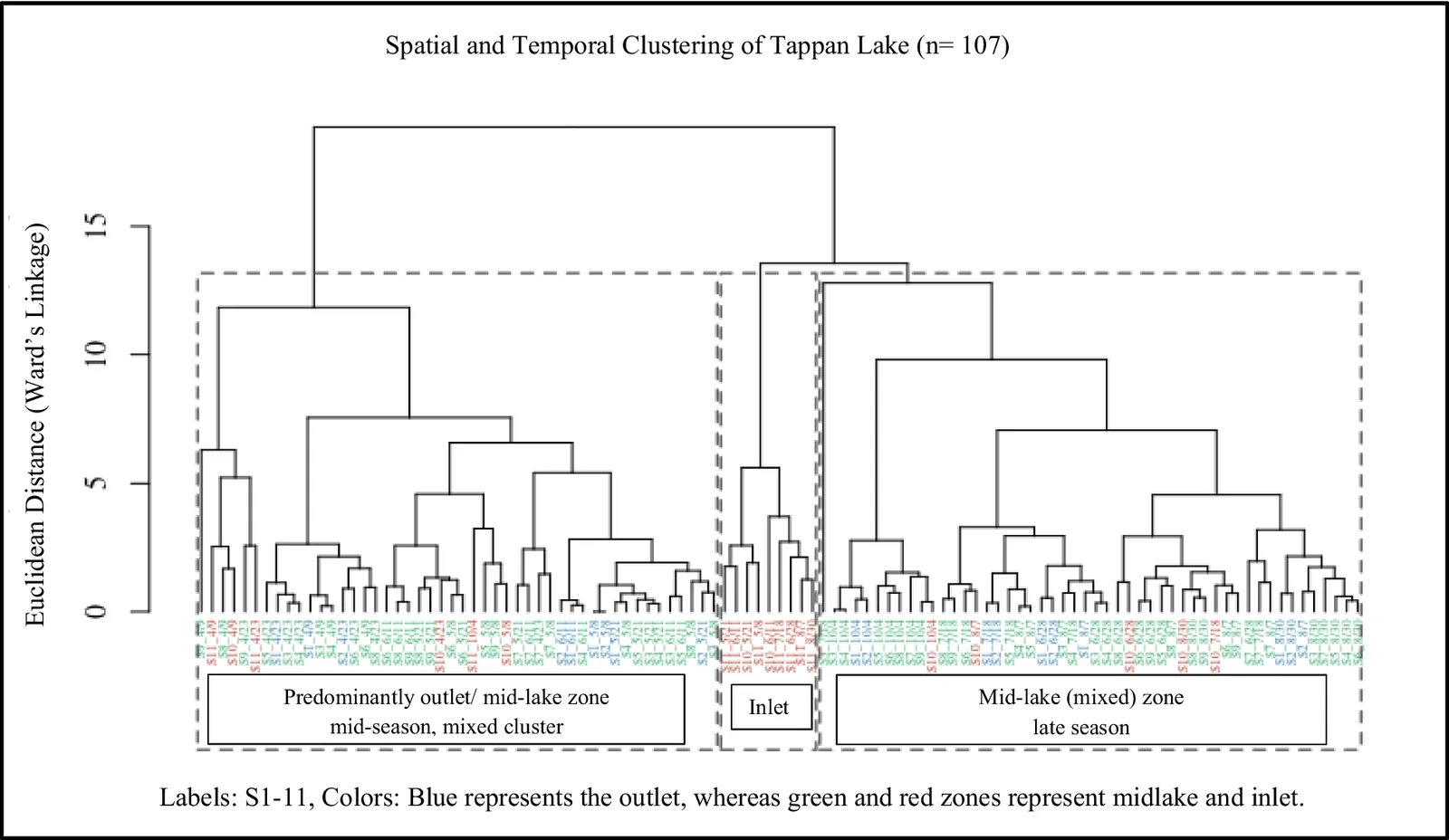
Dendrogram illustrating the hierarchical classification of water quality monitoring stations into ecologically distinct spatial zones and temporal clusters based on Euclidean dissimilarity

Tappan Lake exhibits significant water quality variations across both location and time; these patterns, detailed in Table 5, underscore the dual influence of broader natural processes and site-specific ecosystem dynamics. The mathematical validity of these patterns was confirmed using Agglomerative Hierarchical Clustering (AHC), which identified three primary clusters (Fig. 7). Outlet stations like S1 and S2, which are within proximity to each other, reflect similar trends within the dataset and display consistent temporal patterns. For instance, the temporal warming pattern observed at both sites (Fig. 2a) reflects the expected increase in surface water temperature during spring and summer, even though air‑temperature data were not included for direct comparison. As water temperature increased, both pH and blue‑green algae (BGA) levels rose at the two monitoring locations, reflecting the well‑established temperature‑driven stimulation of photosynthetic activity and cyanobacterial growth (temperature effects, cyanobacterial growth). Similarly, stations S3–S9 within the mid‑lake zone exhibited comparable patterns across key parameters because they experienced similar light availability and hydrodynamic mixing conditions, which tend to homogenize water‑quality responses in central basin areas (mid‑lake mixing).In contrast, S10-S11 at the inlet zone highly distinct patterns (Cluster 2 in Fig. 7), differentiating it from the mid-lake and outlet zone because of watershed input and shallower depth. To sum up, spatial clustering delineated Tappan Lake into three ecologically distinct regions, such as outlet, mid-lake, and inlet stations, which were guided by differing environmental, unique hydrological, and biogeochemical patterns.

**Table 5:** Factor loading and its interpretation across spatial zones and temporal periods

Domain	Subset stations	Dominant PCs (variance %)	Interpretation
**Spatial**	Outlet (S1–S2)	F1 (39%) = Temp (+), BGA (+) F2 (27%) = Turbidity (+), SPC (+), Chl-a (−)	The thermal–chemical axis reflects stable, well-mixed water, small, short-term bloom events F2 captures with moderate biological activity
	Mid-lake (S3–S9)	F1 (46%) = Temp (+), Turbidity (−), pH (+) F2 (23%) = BGA (+), SPC (−), DO (+)	F1 shows strong biological production under stratification F2 represents bloom-driven oxygen depletion
	Inlet (S10–S11)	F1 (49%) = Temp (+), Chl-a (+) F2 (17%) = pH (+), BGA (+), DO (+)	F1 shows nutrient-rich tributary inputs F2 shows algal proliferation coupled with sediment influx
**Temporal**	Spring	F1 (63%) = SPC (+), pH (+), BGA (+) F2 (28%) = Turbidity (+), Chl-a (+), Temp (+)	Well-mixed, oxygen-rich, pre-bloom period with nutrient release after turnover Warmer and more turbid water with moderate biological activity
	Summer	F1 (55%) = Temp (+), Turbidity (+), Chl-a (+), BGA (+) F2 (23%) = pH (+), DO (+)	Temperature and biological indicators reflect the proliferation of an algal bloom High photosynthetic activity causes alkalinity in water, conforming to bloom maturity
	Fall	F1 (62%) = Turbidity (+), DO (+), Chl-a (+) F2 (16%) = Temp (+), pH (+), BGA (+)	Stratification breaks down; oxygen rebounds; reduced algal dominance Possibly late-season cyanobacterial persistence

F1, F2, and F3 represent the first three principal components, capturing the dominant gradients of variability in water quality. Here, “ + ” denotes a positive factor loading, whereas “−” denotes a negative factor loading

Temporally, the spring season was characterized by lower temperatures, BGA, and pH, with higher DO and SPC across all the stations, suggesting oxygen-rich, clear water. The AHC results grouped these spring sampling events into a single homogeneous cluster (Cluster 1 in Fig. 7), confirming high spatial uniformity during this period. As reported earlier, it could be due to the spring runoff-driven road salt input (Diaz, 2008; Ellen Foley, 2023; Kaushal et al., 2018) and early seasonal bloom. Later in the summer, increased temperature, elevated BGA, Chl-a, and pH with lower DO reflected algal bloom and nutrient cycling. This process was driven by summer stratification, facilitating the internal loading by creating anoxic sediment that promotes the release of bound phosphorus, thereby aiding phytoplankton growth (Nürnberg, 1984). Finally, during the late summer months or early fall, recovery began with decreasing temperatures, BGA, pH, and Chl-a with higher DO. In summary, three different temporal groups were clustered, reflecting spring, summer, and fall conditions.

### Permutational multivariate analysis of variance (PERMANOVA)

To quantify the drivers of environmental variability, a Permutational Multivariate Analysis of Variance (PERMANOVA; Anderson, 2001) was employed. The analysis revealed that both spatial and temporal factors significantly structured the data (*p* < 0.001). Spatial zonation emerged as the primary driver, explaining 79% of the total observed variation (*F* = 50.93). This indicates a robust differentiation in water quality characteristics between the inlet, mid-lake, and outlet zones. Temporal fluctuations also exerted a significant, though comparatively lower, influence, accounting for 59% of the total variance (*F* = 21.31). The high *R*^2^ values for both factors suggest that the sampling design effectively captured the dominant environmental gradients within the system, with spatial heterogeneity being the most influential component in defining the reservoir’s limnological profile.

During the spring months, runoff introduced road salt inputs, consequently increasing conductivity and altering the ionic balance in the reservoir, which is consistent with the previous findings (Dhungel et al., 2020). This influx, coupled with the rising temperature and enhanced light availability, further aided early phytoplankton and cyanobacterial growth (Iloba & Akpoyibo, 2019). During the summer months, the lake experienced stratification and mixing events. It demonstrated higher factor loadings of temperatures, BGA, and pH, which acted as the first principal components (F1), reflecting the dominance of temperature-induced bloom in the surface water (epilimnion) and bottom layer (hypolimnion). Conversely, DO exhibited negative loadings, which suggested conditions of hypoxia and limited vertical mixing at the hypolimnion. This indicated clear and stable DO stratification during the summer period. Later in the fall, stratification was interrupted, where decreased temperature and increased DO suggested the redistribution of oxygen and nutrients throughout the depth profile.

## Conclusion

A well-defined stratification pattern, comprising the epilimnion, metalimnion, and hypolimnion, was observed across temporal periods, driven by gradients in temperature, dissolved oxygen, and other key physicochemical properties.

This phenomenon was quantitatively interpreted by PCA using the key water quality parameters and their pattern. The analysis further assisted with the identification of key environmental gradients and exposed the underlying behavior of physicochemical and biological processes in lakes. This investigation revealed a robust spatiotemporal pattern in Tappan Lake’s water quality, where the first two principal components together described approximately 70% of total variance, showing temperature, pH, and BGA as the primary water quality variables associated with principal components. The strong positive correlation between temperature and BGA (*r* = 0.864), coupled with a corresponding negative correlation with DO (*r* = −0.857), created a chance of hypoxia during the summer stratification period. Temporally, the lake shifted from a well-oxygenated spring (DO > 9 mg/L) to a critical hypoxic condition (~ 0.45 mg/L) during summer eutrophication and finally showed signs of recovery in fall (DO≈7–8 mg/L). Spatially, inlet zones were nutrient-enriched and bloom-prone, mid-lake zones experienced strong bloom proliferation with summer stratification and hypoxia, and the outlet stations were relatively stable. Spatially, inlet zones were clustered based on the nutrient enrichment and turbidity level, suggesting influence from watershed inflow. Mid-lake zones exhibited moderate Chl-a and temperature loadings, indicating them to be a temperature-influenced mixing region with extensive biological productivity. Meanwhile, outlet zones experienced higher DO and moderate temperature loadings, suggesting the influence of depth and hydrodynamic flushing of water from the lake. These findings strongly align with the global evidence that the nutrient loading (internal/external) and temporal thermal stratification were the primary factors that elevate cyanobacterial dominance and oxygen depletion. In addition, nitrogen and phosphorus inputs during the fertilizer application season likely contribute to increased algal biomass and bloom development in the lake.

To summarize, watershed-driven nutrient inputs and rising temperatures are the principal variables contributing to the variance of eutrophication in these sorts of lakes, highlighting the critical importance of integrated catchment management and mitigation approaches for summer blooms. The findings of this study provide guidance and actionable insights for policymakers, environmentalists, and local communities to bolster conservation efforts and uphold the long-term ecological integrity of Tappan Lake. Overall, PCA concludes that the water quality of Tappan Lake is governed by stratification and spatial heterogeneity.

## Data Availability

Data will be made available on reasonable request.
